# Prevalence of vessel wall abnormalities and the risk of recurrent vascular events in young patients with stroke

**DOI:** 10.1177/23969873251343828

**Published:** 2025-06-12

**Authors:** Esther M. Boot, Frederick J. A. Meijer, Sjoert Pegge, Sjan Teeselink, Mijntje MI Schellekens, Merel S. Ekker, Jamie I. Verhoeven, Esmée Verburgt, Maikel Immens, Nina Hilkens, Frank-Erik de Leeuw, Anil M. Tuladhar

**Affiliations:** 1 Department of Neurology, Donders Institute for Brain, Cognition and Behaviour, Radboud University Medical Centre, Nijmegen, The Netherlands; 2 Department of Radiology, Donders Institute for Brain, Cognition and Behaviour, Radboud University Medical Centre, Nijmegen, The Netherlands

**Keywords:** Vessel wall imaging, young stroke, ischemic stroke

## Abstract

**Introduction:**

We examined the prevalence and the characteristics of vessel wall (VW) lesions in young stroke patients and their relation to recurrent vascular events. We hypothesize that having VW lesions is associated with an increased risk on recurrent vascular events.

**Patients and methods:**

Single-center prospective study of participants aged 18–50 years, with a transient ischemic attack (TIA) or ischemic stroke, who underwent high-resolution 3T magnetic resonance imaging (HR-MRI) with VW imaging. We included 10 controls with symptoms diagnosed as stroke mimics. The HR-MRI scans were reviewed by two neuroradiologists blinded for clinical information. Follow-up was conducted via telephone interviews. Recurrent vascular events were defined as TIA, cerebral stroke, myocardial infarctions, revascularization procedures, or vascular death.

**Results:**

We included 158 participants (median age: 41.5 years, IQR 33.0–46.4); 75 (47.5%) of whom were women. Of these, 44 (27.8%) participants had 81 VW lesions, primarily characterized by VW enhancement (74.1%). 86.4% of VW lesions were located in the corresponding ischemic territory, and 48.6% showed no MRA abnormalities. Almost half of the VW lesions were found in the rare causes subgroup, while 13.6% of the “cryptogenic” subgroup showed VW enhancement. VW lesions were not significantly associated with an increased risk of recurrent vascular events (HR 2.2, 95% CI: 0.7–6.6).

**Conclusion:**

One in four young stroke patients have VW lesions, which were not related to an increased risk of recurrent vascular events. VW lesions were seen across all TOAST categories and were not specific to one stroke cause. Further research is needed to investigate the diagnostic and prognostic value of VW lesions in young stroke patients.

## Graphical abstract

**Graphical abstract:**
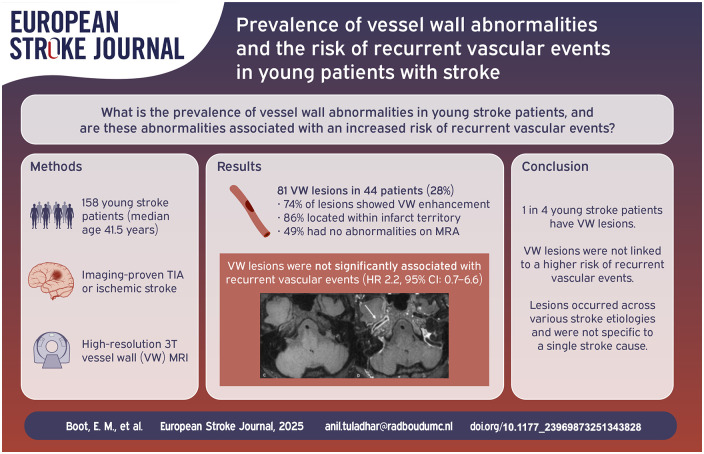

## Introduction

The incidence of stroke worldwide is approximately 15 million events per year,^1^ with 10%–15% of all strokes occurring in patients aged 18–50 years,^2^ so-called young stroke. Over the last few years, the incidence of young stroke has been increasing.^2^ It is of vital importance to determine the underlying etiology of the stroke to start appropriate secondary preventive treatment and to inform the patients about the prognosis and risk of recurrence.^2^ Yet, despite thorough standard diagnostic workup, in up to 25% of young stroke patients, the cause of the stroke remains unknown and participants are still at risk for recurrent stroke with a 20-years cumulative risk as high as 20%.^3,4^

Cervical and intracranial vasculopathies are implicated in 10%–20% of young stroke patients^5^ and visualizing vessel wall abnormalities could clarify stroke causes and prognosis. Standard diagnostic workup such as ultrasound, computed tomography angiography or magnetic resonance angiography (MRA) includes imaging of the vessel lumen to determine the presence of steno-occlusive vasculopathy. However, they are limited in identifying the underlying vessel wall pathology. For example, in non-stenotic vasculopathies,^6^ conventional luminography may show no stenosis, while atherosclerotic plaques can still be present in the vessel wall, due to positive remodeling.^7,8^ Therefore, conventional luminography, as executed in daily practice, may not fully capture all vasculopathies as a cause of stroke.

The vessel wall (VW) of cervical and intracranial arteries can be visualized by high-resolution magnetic resonance imaging (HR-MRI) techniques.^9–11^ HR-MRI can identify VW abnormalities, which can sometimes be subtle and include VW thickening, intramural hematoma (IMH), VW contrast enhancement, and vulnerable plaques.^12^ These VW abnormalities can be related to different type of vasculopathies. For example, VW enhancement (VWE) can be seen in dissection, atherosclerotic arteriopathy, vasculitis, secondary to ischemia, or after mechanical thrombectomy.^10–13^ Earlier research showed the diagnostic impact of HR-MRI on etiology in young stroke patients,^14,15^ however none of these studies focused on the prevalence of VW lesions. The proper adjudication of VW abnormalities may also be important in predicting recurrent vascular events,^16–19^ however there is a paucity of literature on which VW lesion is related to recurrent vascular events in young stroke patients.

The aim of this study is to examine (1) the prevalence of cervical and intracranial VW lesions seen on HR-MRI in a cohort of young ischemic stroke patients and (2) whether these lesions are associated with recurrent vascular events. We hypothesized that young ischemic stroke participants will exhibit less VW lesions compared to stroke in the older population and that these VW lesions are related to an increased risk of stroke recurrence. We would not expect any lesions in our stroke-free controls.

## Patients and methods

### Study population

For this prospective single-center cohort study, we incorporated a subset of consecutive participants from the ODYSSEY study,^20^ as well as an additional subset of consecutive participants who did not meet the eligibility criteria of the ODYSSEY study but did meet our predefined inclusion criteria. Our defined inclusion criteria were: age between 18 and 50 years, diagnosis of an ischemic stroke or transient ischemic attack (TIA) due various stroke etiologies, which was confirmed by a CT or MRI scan, and as a part of the diagnostic work-up, a HR-MRI was typically performed afterward. In some cases with a high suspicion of stroke, HR-MRI was conducted directly, even without CT or MRI confirmation, to avoid the additional burden of multiple scans on vulnerable patients. TIA refers solely to the duration of symptoms, and all participants were required to have an imaging-proven lesion. This specific MRI protocol was only conducted in participants who visited the Radboud Medical Centre. Exclusion criteria were poor quality of the HR-MRI of both the cervical and intracranial vessels. We post-hoc decided to additionally include 10 controls aged 18–50 years, who underwent HR-MRI for symptoms eventually diagnosed as stroke mimics, that is, migraine (*n* = 2), peripheral vestibulopathy (*n* = 2), Transient Headache and Neurologic Deficits With Cerebrospinal Fluid Lymphocytosis (HaNDL) syndrome (*n* = 1), isolated retinal vasculitis (*n* = 1), and functional neurological disorders (*n* = 4). Inclusion took place between January 2016 and September 2021.

### Baseline data collection

Baseline data was systematically collected including stroke characteristics, acute treatment, diagnostic laboratory, and cardiac tests, stroke etiology and vascular risk factors. Vascular risk factors were defined according to the ODYSSEY study protocol.^20^ Stroke etiology was categorized into seven groups based on the definition of the modified TOAST classification; 1a atherothrombotic stroke, 1b likely atherothrombotic stroke, two cardioembolic stroke, three small vessel disease (SVD), four stroke due rare causes, five multiple causes and six cryptogenic.^21^ Crucially, it is important to note that the presence of intramural hematoma (IMH) on HR-MRI vessel wall imaging was considered in assessing the etiology of the dissection, supplementing the conventional luminography techniques of CTA and MRA. For other stroke etiologies, HR-MRI played no role in the assessment of the etiology.

### Follow-up assessment

In the ODYSSEY cohort study, follow-up assessments were conducted via telephone interviews utilizing carefully structured questionnaires annually, starting from participants’ study inclusion and continuing until June 2022. Additionally, a subset of cases where no event had been reported were randomly verified through a review of their medical records. The recurrence of vascular events was assessed with thoroughness, and their confirmation was established by cross-referencing the collected information with the subjects’ medical records, or in cases where such records were unavailable, by consulting the general practitioner. These vascular events comprised cases of cerebral ischemic strokes, TIAs, myocardial infarctions, revascularization procedures, or vascular death (death within 30 days after any of the prior mentioned vascular events).

### MRI protocol

MRI scans were performed on a 3T MRI scanner (Siemens Magnetom Trio, Erlangen, Germany). The standardized imaging protocol included: 3D T1 magnetization prepared rapid gradient echo (MPRAGE; repetition time (TR) 2300 ms, echo time (TE) 2.3 ms, 0.9 mm isotropic voxel), T2 turbo spin echo (TSE; TR 3500 ms, TE 92 ms, slice thickness 5 mm), 3D fluid attenuated inversion recovery (FLAIR; TR 5000 ms, TE 394 ms, 1 mm isotropic voxels), susceptibility weighted imaging (SWI; TR 27 ms, TE 20 ms, slice thickness 3 mm), diffusion weighted imaging (DWI; TR 5000 ms, TE 80 ms, b-values 0 and 1000, slice thickness 5 mm), contrast-enhanced MRA of the extracranial region (TR 2.99 ms, TE 1.09 ms, 1 mm isotropic voxels, admission of 15 ml gadolinium based contrast agent (Dotarem^®^, 0.5 mmol/ml, Guerbet, Villepinte, France), head time of flight MRA (TR 24 ms, TE 3.93 ms, 0.55 mm isotropic voxels) and 3D T1 SPACE VW sequences pre- and postcontrast administration (TR 750 ms, TE 20 ms, voxel size 0.5 × 0.5 × 0.9 mm, slice thickness 0.9 mm, coil elements SP5-8, circle of Willis scanned in an axial plane with acquisition time (TA) of 245 s, extracranial vessels scanned in a coronal plane with TA of 260 s, FoV 231 mm, and 5 cm z-coverage). The average scanning time for this protocol was 43 min.

### Assessment of vessel wall lesions

HR-MRI scans were reviewed by two experienced neuroradiologists (F.J.A.M. and S.P.), who were blinded for clinical information (i.e. clinical symptoms, diagnostics, the final diagnosis, and clinical outcome) and initial prior imaging. The scans were visually reviewed using a standardized protocol, which included scoring of VW features. These features included quality of imaging, fulfillment of imaging protocol, presence of recent and earlier infarction, and location of infarction. The VW features included the presence of an IMH, presence of a plaque, plaque enhancement, presence of VW enhancement and VW thickening and location of these VW features. IMH was defined as hyperintense signal in the VW on the T1-imaging.^22^ Cervical plaque was defined as a hyperintense signal in the VW on the T2 sequence with corresponding hypointense signal on the T1 sequence, with or without corresponding contrast enhancement.^22^ For intracranial plaques, the pre- and postcontrast T-1 imaging sequences were used. VWE was scored by comparing the pre- and postcontrast T1-imaging sequences, with the known pitfalls in consideration.^22,23^ VW thickening was classified as wall thickening on the precontrast T1-imaging, which was scored visually by comparing the adjacent vessel segments.^22,23^ Ten percent of the cohort underwent dual scoring by both raters to calculate interobserver variability using Cohen’s kappa for VWE and VW thickening.

### Statistical analysis

The baseline characteristics include mean and standard deviation for normally distributed continuous data and as median and interquartile range (IQR) for non-normally distributed data. The baseline characteristics between participants with and without VW lesions were compared using the independent t-test for continuous variables and Chi-square/Fisher’s exact test for categorial variables. Furthermore, the characteristics of all the VW lesions were considered in relation to the stroke etiology, MRA and the affected vascular territory. Finally, an age-adjusted Cox regression analysis was performed to investigate whether VW lesions were related to an increased risk of recurrent cerebral ischemic events and any recurrent vascular events. The two-tailed significance level alpha was set at 0.05. Data analysis was carried out with R studio version 1.1463.

## Results

### Baseline characteristics

A total of 158 young stroke participants were included with a median age of 41.5 years (IQR: 33.0–46.4) and 75 (47.5%) were female (Table 1). Of these, 140 participants underwent HR-MRI with VW imaging of both the cervical and intracranial vessels, five participants underwent only VW imaging of the cervical vessels, and 13 participants underwent only VW imaging of the intracranial vessels. Median time between TIA/stroke and MRI was 29.5 days (IQR 5.0–107.5), which did not significantly differ between participants with VW lesions and participants without VW lesions. The median time from TIA/stroke to HR-MRI for patients with dissection was 23 days (IQR: 5–116). For patients with both dissection and VWI abnormalities, the median time was 12 days (IQR: 5–33), while for those without VWI abnormalities, the median time was 33 days (IQR: 6–207). We included 10 stroke-free controls, with a median age of 36.7 years (IQR: 29.3–48.1) and 60% were female.

**Table 1.** Demographic characteristics of 158 young stroke patients.

Variable	All participants (*n* = 158)	Patients with VW lesion (*n* = 44)	Patients without VW lesion (*n* = 114)	*p*-Value
Median age, years (IQR)	41.5 (33.0–46.4)	43.4 (36.5–46.9)	40.6 (31.5–45.6)	0.05
Type event				
TIA	29 (18.4%)	4 (9.1%)	25 (21.9%)	0.07
Stroke	129 (81.6%)	40 (90.9%)	89 (78.1%)	
Female	75 (47.5%)	23 (52.3%)	52 (45.6%)	0.45
Previous stroke/TIA	10 (6.3%)	2 (4.5%)	8 (7.0%)	0.73
Cardiovascular risk factors				
Hypertension^a^	52 (32.9%)	18 (43.9%)	34 (34.7%)	0.31
Diabetes mellitus^b^	1 (0.6%)	0 (0.0%)	1 (1.1%)	1.00
Dyslipidemia^c^	83 (52.5%)	29 (69.1%)	54 (54.6%)	0.11
Smoker at baseline^d^	32(20.5%)	9 (26.5%)	23 (31.1%)	0.63
Obesity^e^	26 (16.5%)	8 (21.1%)	18 (19.2%)	0.80
Illicit drug use^f^	14 (8.9%)	5 (14.7%)	9 (10.7%)	0.42
>2 cardiovascular risk factors^g^	25 (19.2%)	9 (23.7%)	16 (17.4%)	0.41
Cause of stroke				
Atherothrombotic	5 (3.2%)	5 (11.4%)	0 (0%)	**0.001**
Likely atherothrombotic	11 (7.0%)	4 (9.1%)	7 (6.1%)	0.5
Cardio embolic	35 (22.2%)	4 (6.8%)	32 (28.1%)	**0.003**
Small vessel disease	18 (11.3%)	5 (11.4%)	13 (11.4%)	1.00
Multiple	13 (8.2%)	1 (2.3%)	12 (10.5%)	0.11
Rare causes^k^	36 (22.8%)	20 (45.5%)	16 (14.0%)	<**0.001**
Cryptogenic	39 (24.7%)	6 (13.6%)	33 (29.0%)	0.05
NIHSS at baseline (IQR)^h^	3 (1–6)	4 (2–7)	2 (0–5)	0.10
Intra-arterial Thrombectomy^i^	16 (10.1)	5 (11.4)	11 (9.8)	0.78
Time between initial event and HR-MRI, days (IQR)	29.5 (5.0–107.5)	8.0 (4.8–37.3)	43.5 (6.0–114.8)	0.05
Atherothrombotic	8 (7–23)	8 (7–23)	-	NA.
Likely atherothrombotic	5 (4–69)	5 (4.5–37)	8 (2–118)	0.44
Cardio embolic	65 (12–128)	65 (5–204)	57 (12–128)	0.72
Small vessel disease	36.5 (6–131)	146 (26–150)	25 (5–63)	0.18
Multiple	13.5 (8–56)	8 (3.5–28)	15 (12–56)	0.57
Rare causes^k^	8 (3–41)	8 (8–8)	7 (3–76)	0.07
Dissection	23 (5–116)	12 (5–33)	33 (6–207)	0.12
Cryptogenic	58 (6–116,5)	3.5 (2–5)	78 (32–124)	**0.04**
Follow-up duration, years (IQR)^i^	2.5 (1.5–3.8)	3.3 (2.4–4.8)	2.1 (1.4–-3.2)	<**0.001**
Recurrent event^j^	14 (11.6%)	6 (17.1%)	9 (9.3%)	0.22
TIA	5 (35.7%)	2 (33.3%)	3 (37.5%)	
Stroke	8 (57.1%)	3 (50%)	5 (62.5%)	
Myocardial infarction	1 (7.1%)	1(16.7%)	0 (0%)	

VW: vessel wall; IQR: inter quartile range; TIA: transient ischemic attack; NIHSS: National Institutes of Health Stroke Scale.

a 19 missing.

b 29 missing.

c 17 missing.

d 50 missing.

e 26 missing.

f 40 missing.

g 25 participants had more than 2 missing values of the riskfactors and therefore were labeled as missing.

h 7 missing.

i 2 missing.

j Subgroup with 121 participants; 35 with VW lesion, 86 without.

k Consists of: dissection (19); antiphospholipid syndrome (5); drug-induced vasculopathy (6); post-radiation arteriopathy (4); vasculitis (1); partially thrombosed aneurysm (1); cancer (2); hyperleukocytosis (1); hyperhomocysteinemia (2); intoxication with antihypertensive agents (1), Sneddons’ disease (1), factor V leiden (1).

*p* values in bold are statisctically significant.

### Prevalence and characteristics of VW lesions

**Figure 1.**
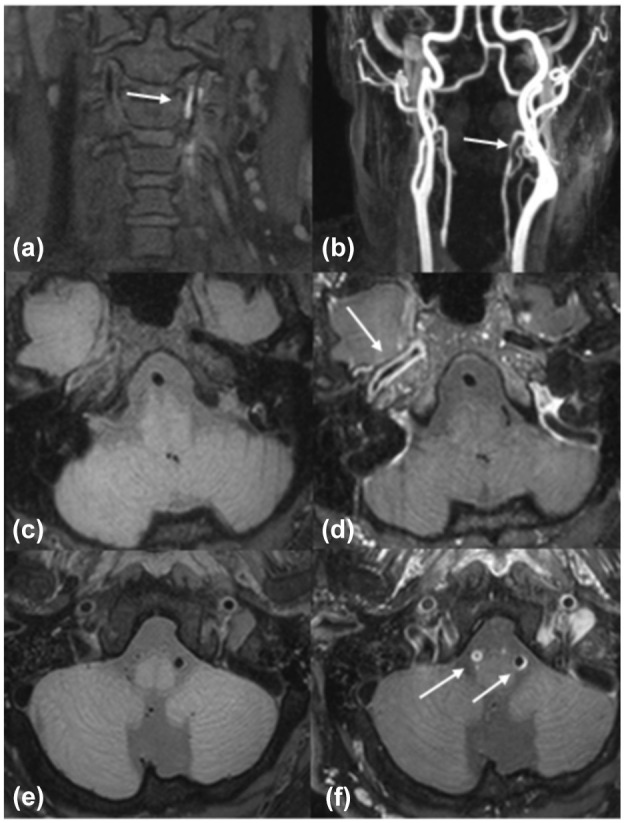
Three cases with different stroke etiologies and VW abnormalities. 1. Patient with an ischemic stroke due to a left vertebral artery dissection. (a) T1 pre-contrast image with a hyperintense region (see white arrow) indicating intramural hematoma. (b) CE-MRA image showing a stenosis in the left vertebral artery (see white arrow). 2. Patient with an ischemic stroke due to large artery atherosclerosis. (c) T1 pre-contrast image with vessel wall thickening of the right ICA. (d) T1 post-contrast image with visible vessel wall enhancement and thickening in the right ICA (see white arrow). 3. Patient with an ischemic stroke due to an intoxication with antihypertensive agents. (e) T1 pre-contrast image. (f) T1 post-contrast image with concentric and eccentric vessel wall enhancement in the vertebral arteries (white arrows).

VW lesions were observed in 44 of 158 participants (27.8%). Among these participants, 11 had only cervical lesions, 26 had only intracranial lesions, and seven had both intra- and cervical lesions (Figure 1). These 44 participants had in total 81 VW lesions, with roughly equal distribution between cervical and intracranial vessels. Multiple VW lesions were seen in 22 participants, with 12 participants showing different VW features in the same vessel. In contrast, no VW lesions were found in our control group. We assessed interobserver agreement for VWE and VW thickening in 16 random participants (10.1%) using the Cohen’s Kappa coefficient. The calculated Cohen’s Kappa for both VWE and VW thickening was 1.0, indicating complete concordance.

Table 2 shows all extra- and intracranial VW lesions and their characteristics. Notably, the majority of VW lesions were localized within the vascular territory corresponding to the ischemic lesion, namely 92.5% of cervical lesions and 80.5% of intracranial lesions. In 48.6% of the cases where VW lesions were present, no abnormalities were found on MRA.

**Table 2.** Vessel wall lesions in cervical and intracranial vessels (*n* = 81).

VW feature	Cervical *n* = 41	Intracranial *n* = 40
*Plaque*	8 (19.5%)	1 (2.5%)
Anterior circulation	8 (100%)	1 (100%)
Corresponding vascular territory	6 (75%)	1 (100)
Plaque enhancement	6 (75%)	0 (0%)
Corresponding abnormality on MRA	2 (25%)^a^	1 (100%)
Corresponding other VW lesion		
IMH	0 (0%)	0 (0%)
Enhancement	4 (50%)	1 (100%)
Thickening	3 (37.5%)	0 (0%)
*IMH*	6 (14.6%)	-
Anterior circulation	3 (50%)	-
Corresponding vascular territory	6 (100%)	-
Corresponding abnormality on MRA	6 (100%)	-
Corresponding other VW lesion		
Plaque	0 (0%)	-
Enhancement	6 (100%)	-
Thickening	2 (33.3%)	-
*Enhancement*	20 (48.8%)	37 (92.5%)
Anterior circulation	12 (60%)	22 (59.5%)
Corresponding vascular territory	18 (90%)	30 (81.1%)
Pattern of involvement		^b^
Concentric	16 (80%)	21 (60%)
Eccentric	4 (20%)	14 (40%)
Pattern of enhancement		^c^
Focal	6 (30%)	18 (54.5%)
Long tract	14 (70%)	15 (45.5%)
Corresponding abnormality on MRA	13 (65%)^a^	12 (34.3%)^b^
Corresponding other VW lesion		
Plaque	2 (10%)	1 (2.7%)
IMH	6 (30%)	0 (0%)
Thickening	5 (25%)	1 (2.7%)
*Thickening*	7 (17.1%)	2 (5%)
Anterior circulation	4 (57.1%)	0 (0%)
Corresponding vascular territory	7 (100%)	2 (100%)
Pattern of involvement	^a^	^a^
Concentric	3 (42.9%)	1 (50%)
Eccentric	3 (42.9%)	0 (0%)
Corresponding abnormality on MRA^a^	3 (33.3%)	1 (50%)
Corresponding other VW lesion		
Plaque	3 (42.9%)	0 (0%)
IMH	2 (28.6%)	0 (0%)
Enhancement	5 (71.4%)	1 (50%)

VW: vessel wall; MRA: magnetic resonance angiography; IMH: intramural hematoma.

a 1 missing.

b 2 missing.

c 4 missing.

### VW lesions and stroke etiology

Participants with VW lesions were more likely to have a stroke classified as atherothrombotic (*p* = 0.001) and rare causes (*p* = 0.001) than participants without VW lesions, and they are less likely to have a stroke due cardioembolic cause (*p* = 0.002). The distribution of VW lesions among different stroke etiologies can be found in the Supplemental table. Multiple VW lesions were related to different etiologies, and the patterns of thickening or contrast enhancement were not restricted to specific causes. VW thickening was observed in different stroke etiologies: one lesion in the likely-atherothrombotic subgroup (concentric pattern), one lesion in the cardio-embolic subgroup (eccentric pattern), and seven lesions in the rare causes subgroup, which were all associated with different types of arteriopathies (cervical dissections, drugs induced arteriopathy, Sneddon’s disease), except one case with hyperhomocysteinemia. VWE was detected in nearly all etiology subgroups, except in the multiple causes subgroup: seven lesions were found in the atherosclerotic subgroup (pattern: five concentric and two eccentric), seven in the likely-atherosclerotic subgroup (pattern: six concentric and one eccentric), two in the cardio-embolic subgroup (pattern: one concentric and one eccentric), 25 in the rare causes subgroup (pattern: 19 concentric and six eccentric), 10 in the SVD subgroup (pattern: five concentric and five eccentric), and six in the cryptogenic subgroup (pattern: one concentric, four eccentric, and one missing pattern). Notably, three plaques were associated with atherothrombotic strokes, two with likely atherothrombotic strokes, two with SVD, one with the cardio-embolic subgroup, and one with arterial dissection. IMH were exclusively found in cervical arterial dissections and not found in other etiologies.

### VW lesions and prognosis

A subset of 121 participants had a follow up with a median follow-up time of 2.5 years (IQR: 1.5–3.8). Fourteen participants (12.4%) experienced a recurrent vascular event including eight with ischemic stroke, five with TIA and one participant with a myocardial infarction (Table 1). Six participants showed VW lesions on HR-MRI at baseline, of which five participants had VW lesions in the vessels supplying the territory of the recurrence. Additionally, these five participants had their recurrent vascular event in the same vascular territory as their index event. In these six participants, 16 VW lesions were observed consisting of five plaques, one IMH, six VWE and four VW thickening. These lesions were found cervical in two participants, intracranial in one participant and both cervical and intracranial in one participant. In participants with VW lesions compared to those without VW lesions, age-adjusted Cox regression analysis did not reveal a statistically significant increased risk of cerebral ischemic recurrence (HR 1.8, 95% CI: 0.6–5.8, *p* = 0.33) or any vascular recurrence (HR 2.2, 95% CI: 0.7–6.6, *p* = 0.17).

## Discussion

In one in four ischemic stroke participants at a young age, we found one or multiple VW lesions on HR-MRI, with VWE emerging as the prevailing abnormality in both cervical and intracranial vessels. In addition, in approximately half of the VW lesions there was no corresponding abnormality on MRA. VW lesions occurred in patients with different stroke etiologies and the patterns of VW thickening and enhancement were not specific to one stroke etiology. Notably, in this study, VW lesions showed no increased risk of recurrent vascular events (HR 2.2, 95% CI: 0.7–6.6).

Since this study is the first to investigate the prevalence of VW lesions in young adults with ischemic stroke, there is a lack of existing literature available for comparison. The observed VW-lesions prevalence of 27.8% is lower than that reported in previous studies conducted in older populations.^24,25^ For example, the Atherosclerosis Risk in Communities (ARIC) cohort study, which focused on participants with cardiovascular disease (mean age: 76.6 ± 5.2 years), reported a prevalence of 36% for intracranial VW lesions, defined as VW thickening, on 3T.^24^ Furthermore, another study involving participants with ischemic stroke or asymptomatic mid cerebral artery stenosis (median age: 64 years, range: 30–84 years) found a 74% prevalence of VWE, of which 90% were in the corresponding ischemic territory. In contrast, the SMART-MR study observed a much higher prevalence of intracranial VW lesions, defined as VW thickening, reaching up to 96%, in participants with cardiovascular disease (mean age: 68 ± 9 years).^26^ These studies differ from ours as they were conducted in population with a higher prevalence of atherosclerotic disease.^27^ Additionally, the discrepancy may also be attributed to differences in MRI field strength, as the SMART-MR employed 7T MRI, which has the potential to detect more lesions compared to 3T MRI.

Numerous studies have indicated that atherosclerosis typically results in eccentric VW thickening and VWE.^12,28^ However, we found one participant with atherothrombotic stroke with concentric VW thickening and concentric VWE. One potential explanation could be that symptomatic plaques, those responsible for symptoms, more likely to have a concentric pattern of VW thickening and VWE compared to non-symptomatic plaques, possibly reflecting extensive neovascularization or inflammatory cell infiltration.^29,30^ Other research has demonstrated that VWE is not always associated with symptomatic disease.^31^ Moreover, our study showed no significant differences between eccentric enhancement and concentric enhancement in relation to symptomatic VW lesions. Furthermore, studies have shown that etiologies in the younger population exhibit different patterns on HR-MRI compared to the older population and do not always align with the specific patterns as suggested earlier.^32,33^

In line with the literature, we found VW lesions in our SVD subgroup, primarily VWE in both cervical and intracranial vessels. In an older population, studies have shown association between VW lesions (defined as VW thickening^26^ or intracranial lesions, of which one-third showed enhancement^34^) and SVD. These results suggests that vascular damage in these patients with cardiovascular risk factors can occur both in large and small vessels simultaneously.

Remarkably, we identified VWE in nearly 14% of participants with cryptogenic stroke, of which 67% of the VWE correspond with the vascular territory of the infarction. This finding is significant in light of previous research that demonstrated how HR-MRI led to the reclassification of predominantly cryptogenic strokes into categories such as atherosclerotic disease or inflammatory vasculopathies.^6,15^ This underscores the potential inadequacy of the TOAST classification in effectively categorizing stroke causes and risk factors among young adults, thereby emphasizing the need for a more comprehensive classification system.^3^

In addition, we observed both concentric and eccentric VWE in participants with post-radiation arteriopathy. Literature on VWE on HR-MRI due to radiation is scarce. One case report described intracranial concentric VW lesions on HR-MRI in a patient with multiple infarcts and a history of whole brain radiation.^35^ One participant with an infectious cerebral vasculitis exhibited concentric VWE over a long tract, which is in line with existing literature.^36^ Together, our findings suggest that VW lesions and the patterns of VWE or VW thickening are not always specific to a particular etiology, underscoring the need for careful examination in young stroke participants. Moreover, HR-MRI may not serve as a widespread screening tool but could be valuable in targeted cases where particular findings hint at conditions such as dissection or vasculitis.

Almost half of the VW lesions, primarily intracranial lesions with VWE, had no corresponding MRA abnormality in our study. This implies that HR-MRI can detect subtle abnormalities otherwise not detected on MRA. In the ARIC study, 10.8% of intracranial plaques (defined as VW thickening) had no detectable stenosis.^24^ This discrepancy is most likely to be related to the low prevalence of symptomatic atherosclerotic disease in our young stroke cohort, as symptomatic atherosclerosis is highly associated with narrowing of the vessel and/or VW thickening.

We did not find an association between VW lesions and the risk of recurrent vascular event at follow-up, despite six out of 14 participants with a recurrent vascular event had VW lesions in the vessels supplying the territory of the recurrence. Previous studies have indicated that certain VW lesions, such as plaque enhancement, VW thickening and VWE, were associated with stroke recurrence in intracranial atherosclerotic disease.^16,37^ However, these patients differed from our participants, as all of them had confirmed intracranial atherosclerotic disease, which could, in addition to the small sample size, explain the discrepancy.

The strength of this single-center study is the use of a clinical HR-MRI protocol, which is reproducible without the need for additional coils or sophisticated sequences, making it comparable to clinical practice. Moreover, HR-MRI scans were reviewed by experienced neuroradiologists, who were blinded for the clinical information and outcome. Additionally, all participants underwent a thorough diagnostic workup and were discussed in multidisciplinary expert meetings to systematically assess the etiological diagnosis.

Several limitations need to be addressed. First, this study, while being one of the largest studies in young stroke patients, had a relatively small sample size, thereby reducing the power and preventing adjustment for all possible confounders. Furthermore, due to these limited sample size, subgroup analyses should be interpreted with caution Second, the tertiary nature of the study center could have led to selection bias, since the case mix may differ from the general population, that is, the more complex cases were referred to our center. Third, as previously noted, the clinical identification of IMH on HR-MRI VW imaging was factored into the diagnostic workup. Consequently, the presence of IMH exclusively in patients with dissection was influenced by this method of categorizing these etiologies. However, other findings found on HR-MRI, that is, VWE as an indicator for early atherosclerosis, were not used in assessing stroke etiology. Fourth, one of the limitations of our study is that we were unable to evaluate recruitment bias, as we did not have data on the number of young stroke patients who refused to participate. Although all eligible patients were included, this limitation may affect the generalizability of our findings. Additionally, the NIHSS scores in our study population were relatively low, indicating that our sample primarily consists of patients with mild strokes. This should be taken into account when considering the generalizability of our results, as it may not fully represent the entire population of young stroke patients. However, the TOAST distribution in our study is comparable to other studies, supporting the representativeness of our findings in that regard. Fifth, blinding the neuroradiologists could have led to difficulties in interpreting the vessel wall, since in clinical practice the radiologist can make comparisons with other imaging modalities or imaging from an earlier phase of the infarction, which also helps to interpret abnormalities of the vessel wall. Sixth, our MRI protocol, mirroring clinical practice, presented certain difficulties in scoring the VW characteristics. For example, we were unable to distinguish different plaque characteristics, such as a thin or ruptured fibrous cap or a lipid-rich necrotic core, in our study due to the lack of dedicated neck coils. Additionally, a drawback of such an MRI series is its substantial scanning time and limited scanning trajectory, making it impractical for concurrent use with a standard MRI/MRA session. Instead, it necessitates targeted scanning specifically directed at the plaque site. These features may indicate vulnerable plaques^13^ and have been identified as independent risk factors for a poor prognosis.^19^ It is important to note that our T1 SPACE sequence of the circle of Willis had a limited field of view, which could lead to the oversight of intracranial VW abnormalities beyond this view. Additionally, our protocol posed challenges in scoring VWT within intracranial vessels, potentially resulting in false-negative results. Conversely, VWE exhibits higher sensitivity and is less prone to being overlooked. Seventh, the study design does not allow for causal inference. VW lesions found in our participants could be an incidental finding and therefore not causal. Finally, the time between the initial event and the HR-MRI varied, which is inevitable in daily clinical practice. Some VW lesions, such as an IMH, VWE, and plaque enhancement, may resolve over time.^38,39^ In our study, we observed a nearly significant association (*p* = 0.05) between time between initial event and HR-MRI and the prevalence of VW lesions, suggesting that some lesions might have resolved at the time of scanning, though caution is needed in interpretation. Given that lesions can disappear, new lesions could also develop, and thus, we cannot definitely establish a direct relationship between the observed lesions and the initial event.

## Conclusion

VW lesions are found in one in four young stroke participants, and HR-MRI is a valuable and promising technique for detecting VW lesions in both cervical and intracranial arteries that may otherwise go unnoticed when only using MRA. Future research is warranted to confirm our findings in a larger cohort and should focus on stratifying the pathological VW in terms of the underlying etiology and the temporal dynamics of the lesions. Also, it is important to assess their added value in clinical practice (i.e. for clinical diagnosis and for individualized treatment), as well as their cost-effectiveness, ideally with a longitudinal study design.

## Supplemental Material

sj-docx-1-eso-10.1177_23969873251343828 – Supplemental material for Prevalence of vessel wall abnormalities and the risk of recurrent vascular events in young patients with stroke Supplemental material, sj-docx-1-eso-10.1177_23969873251343828 for Prevalence of vessel wall abnormalities and the risk of recurrent vascular events in young patients with stroke by Esther M. Boot, Frederick J. A. Meijer, Sjoert Pegge, Sjan Teeselink, Mijntje MI Schellekens, Merel S. Ekker, Jamie I. Verhoeven, Esmée Verburgt, Maikel Immens, Nina Hilkens, Frank-Erik de Leeuw and Anil M. Tuladhar in European Stroke Journal
