# Supplementary material for: Prevalence of vessel wall abnormalities and the risk of recurrent vascular events in young patients with stroke
Source: Eur Stroke J. 2025 Jun 12;10(4):1421–9. doi: 10.1177/23969873251343828 (PMC12165956; doi:10.1177/23969873251343828)
Supplement: sj-docx-1-eso-10.1177_23969873251343828 – Supplemental material for Prevalence of vessel wall abnormalities and the risk of recurrent vascular events in young patients with stroke [file sj-docx-1-eso-10.1177_23969873251343828.docx]

| Supplementary table: Vessel wall lesions per stroke etiology (n=81). | | | | | | |
| --- | --- | --- | --- | --- | --- | --- |
|  | Athero-thrombotic | Likely athero- thrombotic | CE | SVD | Rare causes | Cryptogenic |
| Cervical | **n=4** | **n=4** | **n=2** | **n=6** | **n=25** | **n=0** |
| Plaque, n (%)  enhancement, n (%) | 2 (50)  2 (100%) | 2 (50)  2 (100) | 1 (50)  1 (100) | 2 (33.3)  2 (100) | 1 (4)  0 | -  - |
| IMH, n (%) | 0 | 0 | 0 | 0 | 6 (24) | - |
| Enhancement  concentric/eccentric, n (%)  punctate/long tract, n (%) | 2 (50)  2 (100)/0*  2 (100)/0* | 1 (25)  1 (100)/0  0/1 (100) | 0  0  0 | 4 (66.7)  1 (25)/ 3(75)  0/4 (100) | 13(52)  12 (92.3)/1 (7.7)  4 (30.8)/9 (69.2) | -  -  - |
| Thickening, n (%)  concentric, n (%)/eccentric, n (%) | 0  0/0 | 1 (25)  1 (100)/0 | 1 (50)  0/1 (100) | 0  0 | 5 (20)  2 (50)/2 (50)*** | -  - |
| Corresponding, ischemic territory, n (%) | 0 | 1 (25) | 2 (100) | 6 | 25 (100) | - |
| Corresponding MRA abnormality, n (%) | 3 (75) | 1 (100)** | 0 | 0 | 21 (84) | - |
| Intracranial | **n=6** | **n=6** | **n=2** | **n=6** | **n=14** | **n=6** |
| Plaque, n (%)  enhancement, n (%) | 1 (16.7)  0 | 0  0 | 0  0 | 0  0 | 0  0 | 0  0 |
| IMH, n (%) | 0 | 0 | 0 | 0 | 0 | 0 |
| Enhancement, n (%)  concentric, n (%)/eccentric, n (%)  punctate/long tract, n (%) | 5 (83.3)  3 (60)/2 (40)  3 (60)/2 (40) | 6 (100)  5 (83.3)/1 (16.7)  2 (33.3)/4 (66.7) | 2 (100)  1 (50)/1 (50)  2 (100)/0 | 6 (100)  4 (66.7)/2 (33.3)  2 (50)/2 (50)* | 12 (85.7)  7 (58.3)/5 (41.6)  6 (50)/6 (50) | 6 (100)  1 (25)/3 (75)*  3 (75)/1 (25)* |
| Thickening, n (%)  concentric, n (%)/eccentric | 0  0/0 | 0  0/0 | 0  0/0 | 0  0/0 | 2 (14.3)  1 (100/0*** | 0  0 |
| Corresponding, ischemic territory, n (%) | 5 (83.3) | 5 (83.3) | 2 (100) | 6 (100) | 10 (71.4) | 4 (66.7) |
| Corresponding MRA abnormality, n (%) | 4 ( 66.7) | 2 (40)*** | 1 (50) | 3 (50) | 2 (15.4)*** | 2 (33.3) |
| ** 2 missing, ** 3 missing, *** 1 missing*  *IMH = intramural hematoma ,MRA = magnetic resonance angiography, , CE= cardioembolic, SVD = small vessel disease* | | | | | | |
